# Supplementary material for: Ferritin-Based Single-Electron Devices
Source: Biomolecules. 2022 May 15;12(5):705. doi: 10.3390/biom12050705 (PMC9138424; doi:10.3390/biom12050705)
Supplement: Supplementary file 1 [file biomolecules-12-00705-s001.zip › biomolecules-1716533-supplementary.pdf]

Ferritin-based single-electron devices Supplementary Information

Jacqueline A. Labra-Muñoz,*,†,‡ Arie de Reuver,‡ Friso Koeleman,‡ Martina Huber,† and Herre S. J. van der Zant*,‡

†*Department of Physics, Huygens-Kamerlingh Onnes Laboratory, Leiden University, Niels Bohrweg 2, 2333CA Leiden, the Netherlands*

‡*Kavli Institute of Nanoscience, Delft University of Technology, Lorentzweg 1, 2628 CJ Delft, the Netherlands*

E-mail: j.labramunoz@tudelft.nl; h.s.j.vanderzant@tudelft.nl

Contents

I. Ferritin purity assessment **2**

II. Ferritin size statistics **3**

III. *IV* types (low temperature) **4**

IV. Reference measurements **4**

V. CB experimental data and fits **5**

VI. Charge offset variations **7**

VII. Capacitance and resistance estimates **8**

I. Ferritin purity assessment

Commercial horse-spleen ferritin purchased from Sigma Aldrich (Cat.No. 270-40, Lot: 08E1805) of 54 mg/ml protein concentration was used with no further purification. For the conductance measurements, the ferritin solution was diluted by a factor of 200 using a buffer solution (0.15 M NaCl and 10 mM Tris, pH 8.0, 0.1% sodium azide). The final solution has a protein concentration of 270 μg/ml with >95% purity as assessed by sodium dodecyl sulfate–polyacrylamide gel electrophoresis (SDS-PAGE, see Fig. S1c). The strong presence of ferritin monomers is detected (strong band around 500 kDa). A less intense band of multimers or ferritin aggregates are detected as well (weaker bands around 1000-1200 kDa). Dynamic light scattering (DLS) confirms these findings and shows the peak of greatest intensity centered around 12 nm (Fig. S1a). Two other peaks are present, centered at 265 and 2780 nm, attributed to the appearance of contaminants when handling the sample, e.g., dust. Note that these 2 peaks have a mass that is smaller than 0.5% of the total mass, which indicates that more than 99.5% of the total mass is attributed to ferritin (single particles and aggregates).

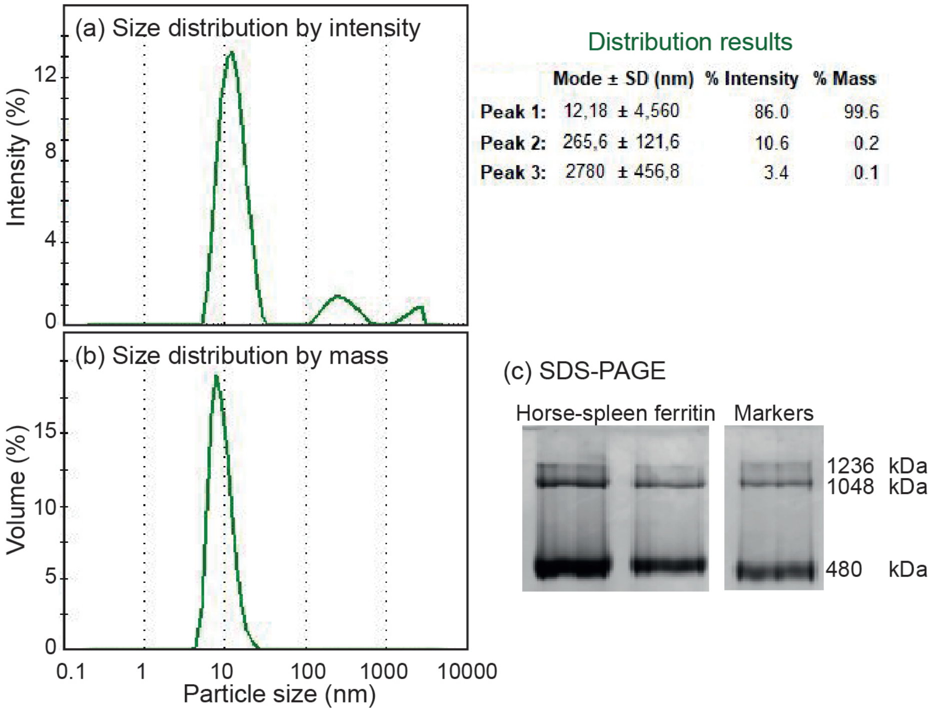

Figure S1: Purity assessment of horse-spleen ferritin. (a,b) Dynamic light scattering showing the particle size distribution: one main Gaussian peak with μ=12.2 nm and σ=4.5 nm. (c) Non-denaturing polyacrylamide gel electrophoresis of horse-spleen ferritin stained with Coomassie. The strong band of 500 kDa agrees with ferritin monomers (24-mer protein) while the weaker bands of 1.048 and 1.236 kDa correspond to ferritin multimers (dimers/trimers) or aggregates of ferritin protein. Markers refers to NativeMark Unstained Protein Standard (Cat. No. LC0725).

II. Ferritin size statistics

The ferritin solution, used in our conductance experiments, was inspected by transmission electron microscopy (TEM). Figure S2a shows the ferritin cores (black dots). A home-made Matlab code was written to automatically detect the cores, by approximating them to circles using the Matlab function *regionprops*; preprocessing of 9 images was performed to enhance the detection, considering the *adapthisteq* function for contrast enhancing. Below 4 nm, the algorithm doesn't distinguish between particles and background noise. The size distribution obtained for 1502 detected ferritin cores is shown in figure S2b. To visualize the organic shell, the ferritin solution was stained with uranyl acetate (2% solution). Figure S2c depicts cores and organic shells; notice that not all cores are visible after staining. Their visibility depends on the orientation of the shells. Figure S2d shows the shell-size statistics of 395 particles whose diameters were measured manually.

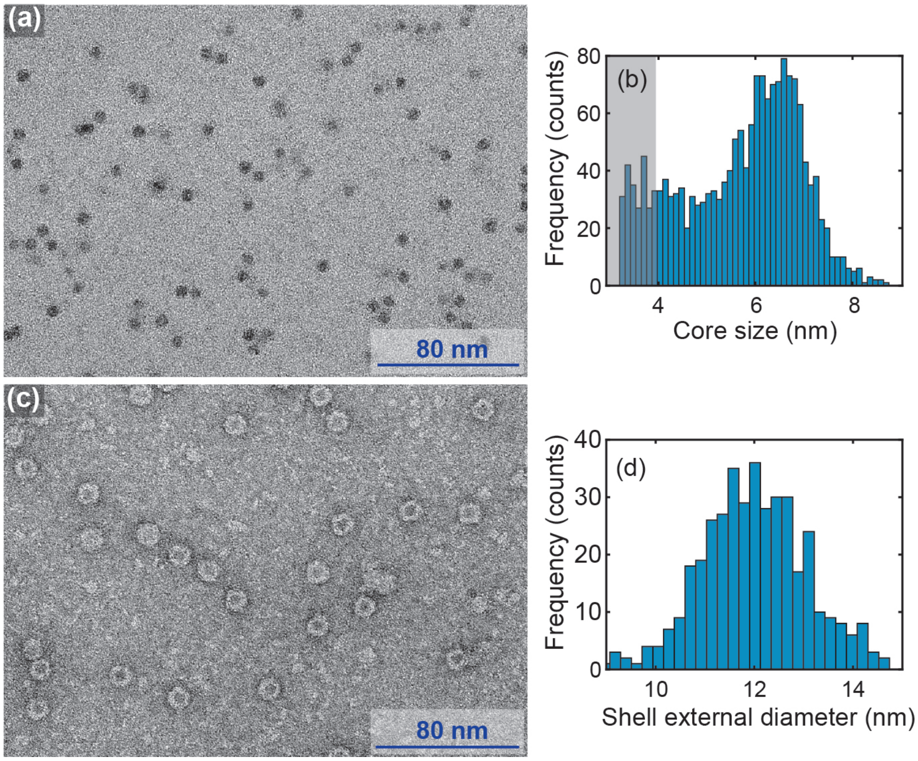

Figure S2: Transmission electron microscopy (TEM) characterization of horse-spleen ferritin. (a) TEM image of horse-spleen ferritin cores. (b) Core-size distribution obtained by the automatic detection of 1502 ferritin cores; the grey coloured area represents the noise region, where it is no possible to discriminate ferritin from image noise. Cores below 4 nm are not accurately identified. (c) Stained-SEM image of horse-spleen ferritins allowing the protein shell visualization. (d) Shell-diameter distribution obtained by manual inspection of 395 ferritin particles.

III. *IV* types (low temperature)

After ferritin deposition, four different IV curve types were obtained, shown in figure S3. Normally, around 90-95 % devices remained open (red dots; left panel); 0-10 % devices show a conductive linear behaviour (green dots; left panel); 3-8 % show Coulomb-blockade-like features (blue dots; middle panel); some devices (0-1 %) show tunneling-like IV characteristics (purple dots; right panel).

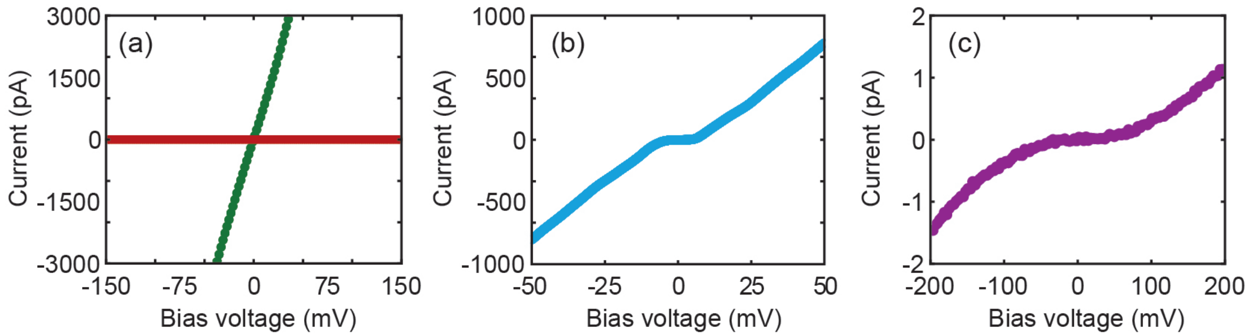

Figure S3: Examples of the four types of experimental current-voltage (IV) characteristics measured after deposition of ferritin at 4.2 K; IVs are recorded by sweeping the voltage from negative to positive values, and vice versa. Note, the absence of hysteresis. (a) Device that remains open (red dots) and a device that shows a highly conductive linear behaviour (green dots). (b) Device that shows Coulomb-blockade-like features. (c) Device that shows tunnelling-like behaviour.

IV. Reference measurements

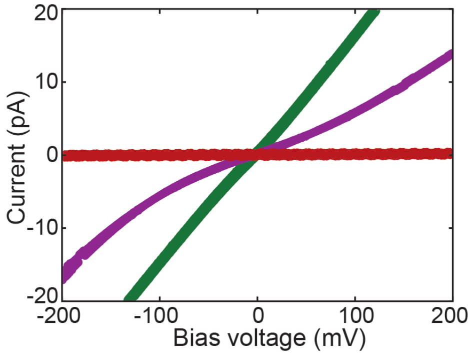

Figure S4: Reference measurements, measured at 4.2 K with just the buffer solution without ferritin (see Ferritin purity assessment). The buffer has been deposited on two different chips (A, B): $3-4$ μl of buffer was deposited three times on each device. After the 3rd buffer deposition, 100% of devices from chip A (5 devices) and 77% of the devices from chip B (10 devices) remained open (red dots). The 33% of the devices on chip B show either tunnelling (purple dots, 2 devices) or linear behaviour (green dots, 1 device). No Coulomb-blockade-like IVs were measured in any device on either chip.

V. CB experimental data and fits

22 different devices showing CB-like features were measured (blue dots, Fig. S5). For all of them, CB fits were performed (black dashed lines), by using the parameters that are shown in table S1. The name of a device is composed of a letter and a number. The letter indicates a specific electrode pair of a chip (source-gap-drain). The number refers to the CB version that was detected: in some cases an abrupt change in the *IV* shape and specifically in the conductivity was detected after any of the following 3 processes:

- thermal cycle: warming it up until 250 K and then cooling it down again to 4.2 K;
- voltaic cycle: after applying more than 0.5 V;
- temporal cycle: random spontaneous change, e.g., hours, days, or after 2 weeks.

This means that devices D1 and D2 are two different CB realizations measured with the same electrode pair.

Table S1: Coulomb blockade simulations parameters, used to generate the 22 simulated *IV*s depicted in Fig. S5. C_1 and C_2 are the junction tunnel capacitances on the left and right sides, respectively. R_1 and R_2 are the tunnel resistances on the left and right sides, respectively. Q_0 is the offset charge. T is the temperature, taken to be the temperature measured close to the sample.

Device	C_1 (aF)	C_2 (aF)	R_1 (GΩ)	R_2 (GΩ)	Q_0 (e)	T (K)
A1	28.0	34.7	0.0004	0.084	0.15	4.2
B1	20.0	30.0	0.2	0.14	-0.15	4.2
C1	1.1	1.1	7.5	7.0	0.24	80.0
D1	12.0	12.0	5.5	5.5	-0.35	4.2
D2	12.0	12.0	0.00042	0.022	-0.545	5.0
E1	9.5	9.2	0.0178	0.0178	-0.12	4.2
E2	4.5	6.5	0.65	0.3	-0.2	5.0
E3	6.5	4.5	0.0031	0.064	-0.15	4.2
F1	4.3	3.4	0.04	0.052	0.75	5.0
G1	0.42	0.42	37.0	35.0	0.08	16.0
H1	0.8	1.7	22.0	24.0	-0.01	0.8
I1	0.8	8.73	0.0086	0.0382	-0.062	4.2
J1	2.6	1.5	15.0	7.0	0.37	1.8
J2	2.6	1.5	32.0	10.5	0.05	1.8
K1	0.17	0.95	7.7	9.8	0.3	3.0
L1	0.7	1.0	4.8	19.8	0.3	4.5
M1	1.2	1.5	8.3	3.8	-0.22	4.5
M2	6.0	7.0	4.5	5.6	-0.37	4.2
N1	0.7	1.0	0.6	0.5	0.05	4.2
O1	3.0	3.0	0.09	0.08	-0.15	4.2
O2	1.1	1.8	4.0	4.0	0.03	4.2
P1	0.5	1.2	10.0	5.0	0.16	4.2

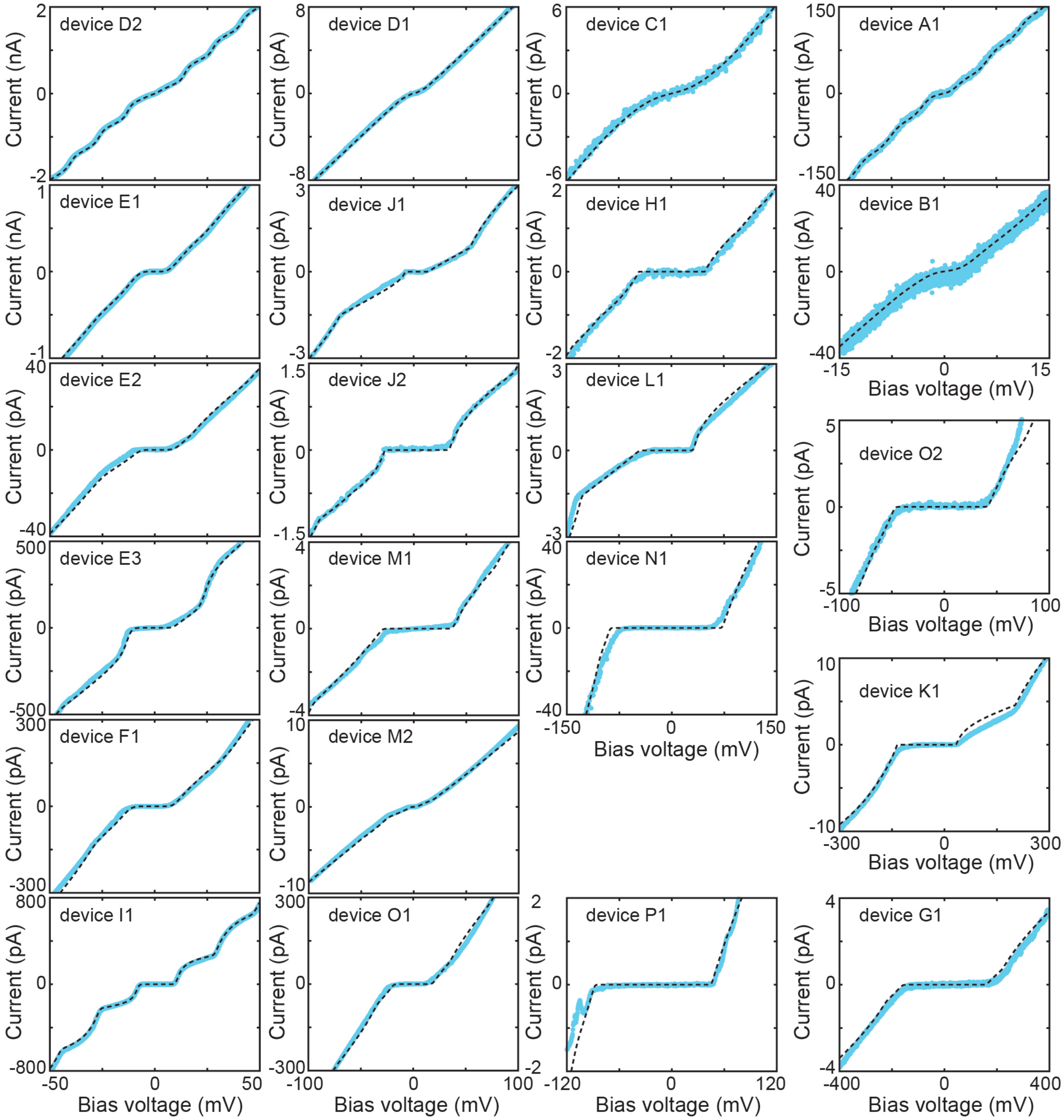

Figure S5: Low-temperature experimental current-voltage characteristics (blue dots) and the corresponding calculated curves using the orthodox Coulomb blockade model (black dashed lines) acquired on the 22 devices displaying Coulomb blockade-like features. The fit parameters and the temperature at which the measurement was performed are collected in Table S1.

VI. Charge offset variations

All 22 devices showed charge offset, Q_0, changes. In some cases this occured after a day of stable measurements, in other devices this happened within a day or a few hours. Figure S6 shows two different devices displaying Q_0 changes within the same day.

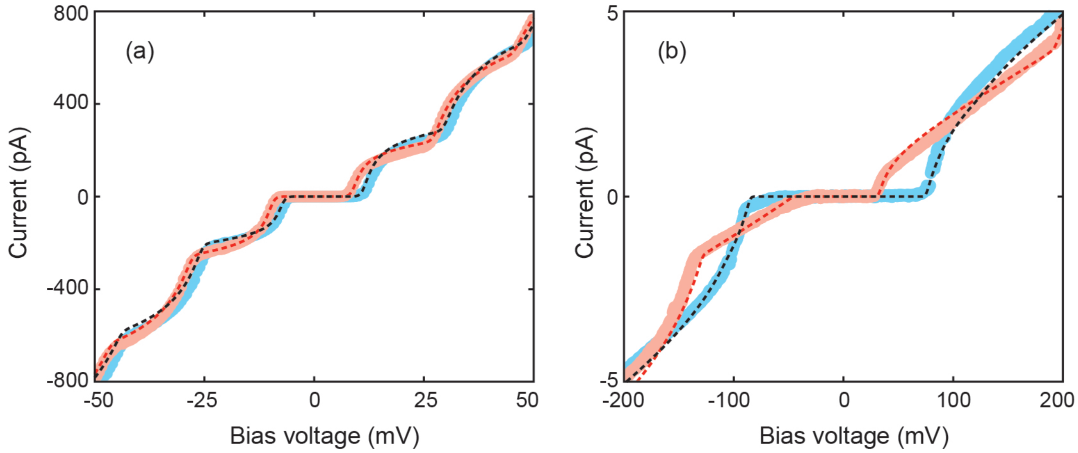

Figure S6: Experimental current-voltage (IV) characteristics (coloured dots) and the corresponding calculated curves using the Coulomb blockade model (dashed lines) acquired on the same device, at different times (same day). (a) Device I1: Q_0 changes from 0.02e (red curve) to -0.11e (blue curve). (b) Device L1, Q_0 changes from 0.3e (red curve) to 0.03e (blue curve). All other parameters remained the same.

VII. Capacitance and resistance estimates

A crude model to estimate the capacitances of ferritin particles is based on two parallel plate capacitors connected between the ferritin mineral core and the two electrodes, on either side (C_1, C_2). These capacitances are expressed as

$$C_{1,2} = \epsilon_r \cdot \epsilon_0 \cdot A/d, \tag{1}$$

where ϵ_0 is the vacuum permittivity and ϵ_r is the relative permittivity of the ferritin organic shell. Laghaei et al., 2014 assigned 20 to the ϵ_r of H-chain ferritin. In addition, Li et al., 2013 modelled ϵ_r in proteins and established that it varies between 6 and 30, for inner and outer regions of the proteins. For our estimates, we consider ϵ_r to be between 10 and 20. Lastly, A is the contact area, and d is the distance between the plates. The total capacitance is denoted as $C = C_1 + C_2$.

In the Coulomb blockade (CB) model, the resistance (R) of an individual ferritin particle trapped in the gap formed in between the source and drain eelctrodes, can be considered as composed of two tunnel resistances in series, one from the source electrode to the core and one from the core to the drain electrode, i.e., $R = R_1 + R_2$. Each of these resistances can be expressed as

$$R_{1,2} = R_0 \exp(\beta d), \tag{2}$$

where R_0 is an effective contact resistance, d is the thickness of the dielectric film (same as in equation 1), and β is the exponential distance decay factor.

With these two expressions we now calculate the capacitances and resistances for 4 different limit cases (see Fig. S7). For simplicity, the ferritin particle is depicted as a cube, but the reasoning is analogous for a spheric particle.

Capacitance estimates

Case I: symmetric in between the electrodes. This case (Fig. S7a) corresponds to the upper bound estimate for the capacitance, since it considers the contact area, A, to be maximized (8 x 8 nm^2). d is considered to be the ferritin-shell thickness ($d_s \sim 2$ nm). The capacitance estimates are shown in table S2.

Table S2: Total capacitance values (C) estimated by assuming a parallel-plate-capacitor model, for case I. $C = C_1 + C_2$ with $C_1 = C_2$ and $d = d_s$.

A (nm^2)	d (nm)	ϵ_r	C (aF)
8 x 8	2	10	5.7
8 x 8	2	20	11.3

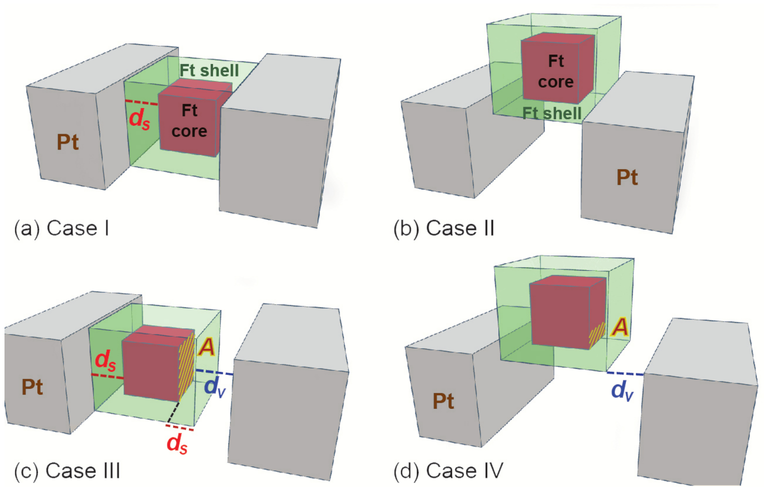

Figure S7: Schematic of the limiting cases of the ferritin parallel-plate capacitor (C) and resistance (R) estimates. The contact area, the protein shell thickness and the vacuum distance are represented by the letters A, d_s, d_v, respectively. (a) Case I: the ferritin particle is connected symmetrically to both electrodes while maximizing the contact area. (b) Case II: ferritin is symmetrically connected to the electrodes while minimizing the contact area. (c) Case III: the ferritin particle is connected asymmetrically to both electrodes (vacuum space in between) while maximizing the contact area. (d) Case IV: the ferritin particle is connected asymmetrically to both electrodes (vacuum space in between) while minimazing the contact area.

Case II: symmetric on top of the electrodes. In this scenario (Fig. S7b), the ferritin particle is no longer in between the electrodes, but rather, on top of them. This results in A being small and we take 1 x 8 nm^2 as the minimal area; d is the thickness of the ferritin shell ($\sim$ 2 nm). The total estimated capacitances are shown in table S3.

Table S3: Total capacitance values (C) estimated by assuming a parallel plate capacitor model, for case II. $C = C_1 + C_2$ with $C_1 = C_2$ and $d = d_s$.

A (nm^2)	d (nm)	ϵ_r	C (aF)
1 x 8	2	10	0.71
1 x 8	2	20	1.42

Case III: asymmetric in between the electrodes. In this situation (Fig. S7c), the gap between the electrodes is bigger than the ferritin diameter, resulting in an asymmetric coupling to the electrodes. One side of the ferritin particle makes contact with one of the electrodes, but there is some space in between the particle and the other electrode. The capacitance of the latter side of the ferritin (C_2) can be estimated by considering a parallel-plate capacitor made of two different dielectrics (the ferritin shell and vacuum), which is effectively two capacitors in series of capacitances $\epsilon_r \epsilon_0 A/d_s$ and $\epsilon_0 A/d_v$, expressed as

$$C_2 = \frac{\epsilon_r \epsilon_0 A}{d_s + \epsilon_r d_v}, \tag{3}$$

where d_v is the distance between the ferritin shell and the electrode. Table S4 shows the estimated capacitances.

Table S4: Total capacitance values (C) estimated by assuming a parallel plate capacitor model, for case III. $C = C_1 + C_2$ with $C_1 > C_2$.

A (nm^2)	d_s (nm)	d_v (nm)	ϵ_r	C_1 (aF)	C_2 (aF)	C (aF)
8 x 8	2	1	10	2.8	0.5	3.3
8 x 8	2	1.5	10	2.8	0.4	3.2
8 x 8	2	1	20	5.7	0.5	6.2
8 x 8	2	1.5	20	5.7	0.3	6.0

Case IV: asymmetric on top of the electrodes. In this case (Fig. S7d), the ferritin particle is on top of one electrode (as in case II), but there is a space between the particle and the other electrode (asymmetric barriers) as in case III. To estimate the total capacitance we can combine the strategies followed on cases II (A = 1 x 8 nm^2) and III. The estimated capacitances are shown in table S5.

Table S5: Total capacitance values (C) estimated by assuming a parallel plate capacitor model, for case IV. $C = C_1 + C_2$ with $C_1 > C_2$.

A (nm^2)	d_s (nm)	d_v (nm)	ϵ_r	C_1 (aF)	C_2 (aF)	C (aF)
1 x 8	2	1	10	0.35	0.06	0.41
1 x 8	2	1.5	10	0.35	0.05	0.40
1 x 8	2	1	20	0.7	0.07	0.77
1 x 8	2	1.5	20	0.7	0.05	0.75

Resistance estimates

Decay β values, at 80 K for different proteins, have been reported to vary from 0.19 Å^{-1} for holo-azurin, to 0.33 Å^{-1} for apo-azurin: azurin without Cu (Amdursky et al., 2014). For our estimates we consider the average value of $\beta = 0.26$ Å^{-1}. To estimate the contact resistance we use the results of a measurement on a single (holo)azurin. A resistance value of 1.8 GΩ has been reported for this protein. Considering a contact area of 9 nm^2, a length of 3.3 nm (Amdursky et al., 2014), and a β of 0.19 Å^{-1} in equation 2, the contact resistance (R_0) equals 3.4 MΩ. We assume that the contact resistance is inversely proportional to the contact area, so the contact resistance per nm^2 is $R_0^* = 30.6$ MΩ$\cdot$ nm^2. With these numbers for β and R_0^* we now calculate the resistance values for the four cases considered before.

Case I: symmetric in between the electrodes. This case (Fig. S7a) corresponds to the lower bound estimate of the total resistance, where A is considered to be 8 x 8 nm^2; therefore $R_0 =$

0.48 MΩ. The resistances are symmetric, i.e., $R_1 = R_2$. The total resistance $R = R_1 + R_2$ is shown in table S6.

Table S6: Resistance values estimated assuming two tunnel resistances in series, for case I. $R = R_1 + R_2$, with $R_1 = R_2$.

A (nm^2)	R_0 (MΩ)	β (Å^{-1})	R (GΩ)
8 x 8	0.48	0.26	0.17

Case II: symmetric on top of the electrodes. In this case (Fig. S7b), the contact area A is minimum (1 x 8 nm^2), therefore $R_0 = 3.83$ MΩ. The table with the estimated resistances for this scenario are depicted in table S7.

Table S7: Resistance values estimated assuming two tunnel resistances in series, for case II. $R = R_1 + R_2$, with $R_1 = R_2$.

A (nm^2)	R_0 (MΩ)	β (Å^{-1})	R (GΩ)
1 x 8	3.83	0.26	1.39

Case III: asymmetric in between the electrodes. In this situation (Fig. S7c), the gap between the electrodes is bigger than the ferritin size, resulting in, e.g., an extra space between the ferritin and one of the electrodes. We can consider this extra space (d_v) as a third tunnel resistance (in vacuum) connected in series to the others. For simplicity, the vacuum space is considered as a prolongation of the ferritin shell, i.e., it exhibits the same $\beta-$decay parameter. This estimation yields a resistance that is smaller than it would be if vacuum was considered with a barrier of 5 eV. The resistance estimates are depicted in Table S8.

Table S8: Resistance values estimated assuming two tunnel resistances in series, for case III. $R = R_1 + R_2$, with R_1 the resistance due to the protein shell only; R_2 is the resistance due to the protein shell and the vacuum space, considered as an extension of the organic shell.

A (nm^2)	R_0 (MΩ)	β (Å^{-1})	d_v(nm)	R_1 (GΩ)	R_2 (GΩ)	R (GΩ)
8 x 8	0.48	0.26	1	0.09	1.17	1.26
8 x 8	0.48	0.26	1.5	0.09	4.30	4.39
8 x 8	0.48	0.26	2	0.09	15.77	15.86

Case IV: asymmetric on top of the electrodes. This is scenario (Fig. S7d) is a combination of cases II and III, i.e., the contact area is minimum (1 x 8 nm^2) and the vacuum space is treated is a prolongation of the organic protein barrier (d_v). The total resistance estimates are presented in table S9.

Table S9: Resistance values estimated assuming two tunnel resistances in series, for case IV. $R = R_1 + R_2$, with R_1 the resistance due to the protein shell only; R_2 is the resistance due to the protein shell and the vacuum space, considered as an extension of the organic shell.

A (nm^2)	R_0 (MΩ)	β (Å^{-1})	d_v(nm)	R_1 (GΩ)	R_2 (GΩ)	R (GΩ)
1 x 8	3.83	0.26	1	0.70	9.35	10.05
1 x 8	3.83	0.26	1.5	0.70	34.30	35.00
1 x 8	3.83	0.26	2	0.70	125.85	126.55

Discussion

Considering the four different cases, the ratio between the minimum and maximum estimated capacitances ($C_{min} = 0.4/C_{max} = 11.3$) is 0.035 when taking the variation of ϵ_r between 10 and 20 into account. From the CB fits to the experimental data, C_{min}/C_{max} is 0.01, which is somewhat lower than our estimate. The maximum estimated capacitance (11.3 aF) is about 5 times lower than the maximum experimental capacitance (62.7 aF). A possible explanation to account for this difference is to consider a barrier thickness thinner than 2 nm, which is plausible from our shell TEM image analysis (Fig. S2d) where thickness variations of ± 1 nm are detected. The maximum capacitance (case I) obtained for a shell thickness of 1 nm is 22.6 aF, which is 2.7 times lower than the maximum experimental capacitance. Another aspect to consider is that our assumed dielectric constant might be different for ferritin. It has been reported that ϵ_r can be 80 for aqueous regions close to ferritin (Li et al., 2013). If we consider the previous example, but with $\epsilon_r = 55$ instead, a maximum capacitance of 62.3 aF is obtained. Note that only 2 out of 25 experimental capacitances are greater than 24 aF, so these cases are rather isolated events.

The ratio between the minimum and maximum estimated resistances (R_{min}/R_{max}) is 10^{-3}. From the CB fits to the experimental data, R_{min}/R_{max} is $2 \cdot 10^{-4}$, which is a factor of 5 lower. The maximum experimental resistance is within the range of our estimates. However, the minimum experimental resistance (0.02 GΩ) is lower than our minimum estimate (0.17 GΩ). One possible reason is that β is smaller than what we assumed. For example, if we consider $\beta = 0.21$ Å^{-1} instead, R_{min} (case I) is 0.06 GΩ, which is in the same order of magnitude as the smaller experimental resistance.

References

Laghaei, R.; Kowallis, W.; Evans, D. G.; Coalson, R. D. Calculation of Iron Transport through Human H-chain Ferritin. *The Journal of Physical Chemistry A* **2014**, *118*, 7442–7453.

Li, L.; Li, C.; Zhang, Z.; Alexov, E. On the Dielectric "Constant" of Proteins: Smooth Dielectric Function for Macromolecular Modeling and Its Implementation in DelPhi. *Journal of Chemical Theory and Computation* **2013**, *9*, 2126–2136.

Amdursky, N.; Marchak, D.; Sepunaru, L.; Pecht, I.; Sheves, M.; Cahen, D. Electronic Transport via Proteins. *Advanced Materials* **2014**, *26*, 7142–7161.
